# Pax3 expression enhances PDGF-B-induced brainstem gliomagenesis and characterizes a subset of brainstem glioma

**DOI:** 10.1186/s40478-014-0134-6

**Published:** 2014-10-21

**Authors:** Katherine L Misuraca, Kelly L Barton, Alexander Chung, Alexander K Diaz, Simon J Conway, David L Corcoran, Suzanne J Baker, Oren J Becher

**Affiliations:** Graduate Program in Molecular Cancer Biology, Duke University, Durham, NC USA; Division of Pediatric Hematology-Oncology, Duke University Medical Center, Durham, NC USA; Preston Robert Tisch Brain Tumor Center, Duke University Medical Center, Durham, NC USA; Integrated Biomedical Sciences Program, University of Tennessee Health Science Center, Memphis, TN USA; Department of Developmental Neurobiology, St. Jude Children’s Research Hospital, Memphis, TN 38105 USA; Developmental Biology and Neonatal Medicine Program, HB Wells Center for Pediatric Research, Indiana University School of Medicine, Indianapolis, IN 46202 USA; Institute for Genome Sciences and Policy, Duke University, Durham, NC USA; Department of Pathology, Duke University Medical Center, Durham, NC USA; Department of Pediatrics, Duke University Medical Center, 450 Research Drive, Durham, NC 27710 USA

**Keywords:** Brainstem Glioma, Pax3, DIPG, ACVR1

## Abstract

High-grade Brainstem Glioma (BSG), also known as Diffuse Intrinsic Pontine Glioma (DIPG), is an incurable pediatric brain cancer. Increasing evidence supports the existence of regional differences in gliomagenesis such that BSG is considered a distinct disease from glioma of the cerebral cortex (CG). In an effort to elucidate unique characteristics of BSG, we conducted expression analysis of mouse PDGF-B-driven BSG and CG initiated in Nestin progenitor cells and identified a short list of expression changes specific to the brainstem gliomagenesis process, including abnormal upregulation of paired box 3 (*Pax3*). In the neonatal mouse brain, Pax3 expression marks a subset of brainstem progenitor cells, while it is absent from the cerebral cortex, mirroring its regional expression in glioma. Ectopic expression of Pax3 in normal brainstem progenitors *in vitro* shows that Pax3 inhibits apoptosis. Pax3-induced inhibition of apoptosis is p53-dependent, however, and in the absence of p53, Pax3 promotes proliferation of brainstem progenitors. *In vivo*, Pax3 enhances PDGF-B-driven gliomagenesis by shortening tumor latency and increasing tumor penetrance and grade, in a region-specific manner, while loss of Pax3 function extends survival of PDGF-B-driven;p53-deficient BSG-bearing mice by 33%. Importantly, Pax3 is regionally expressed in human glioma as well, with high *PAX3* mRNA characterizing 40% of human BSG, revealing a subset of tumors that significantly associates with *PDGFRA* alterations, amplifications of cell cycle regulatory genes, and is exclusive of *ACVR1* mutations. Collectively, these data suggest that regional Pax3 expression not only marks a novel subset of BSG but also contributes to PDGF-B-induced brainstem gliomagenesis.

**Electronic supplementary material:**

The online version of this article (doi:10.1186/s40478-014-0134-6) contains supplementary material, which is available to authorized users.

## Introduction

Brainstem Glioma (BSG) is a brain tumor that arises anywhere in the brainstem, and is seen predominately in children. Although BSG accounts for only 15-20% of all pediatric brain tumors, it is the leading cause of death for children with brain tumors [1]. The majority of BSG (80-85%) are high grade, diffuse, and are located in the pons—these are also known as Diffuse Intrinsic Pontine Glioma (DIPG) and have an overall survival of less than one year [1]. The term BSG hereafter will refer to high grade BSG or DIPG. Conventional focal radiation therapy remains the standard-of-care for these tumors, with no documented additional benefit for any alternative treatment [2].

Genetic alterations commonly found in BSG include gains and/or activating mutations in platelet-derived growth factor receptor alpha (*PDGFRA*), amplification of genes involved in the receptor tyrosine kinase-Ras-phosphoinositide 3-kinase signaling pathway or cell cycle regulation, and a K27M mutation of histone H3.3 or 3.1 [3-9]. Recently, mutations in the *ACVR1* gene have been discovered in 20-32% of cases [10-13], as well as mutations in the *PPM1D* gene [11,14]. The most frequent tumor suppressor lost is *TP53*, with *INK4A*-*ARF* less commonly absent at the genomic level but possibly silenced through alternative mechanisms [3,6,15,16]. Previous work on BSG has characterized distinct subsets of the disease, including mesenchymal and oligodendroglial [5], N-Myc and Hedgehog [17], MYCN, silent, and H3-K27M [10] or H3-K27M and wildtype [6]. Together, these classifications highlight the heterogeneity of this disease and the likelihood that effective treatment will require an understanding of the mechanisms driving the growth of each individual subtype.

In addition to the heterogeneity within pediatric BSG, increasing evidence suggests that this disease is biologically distinct not only from adult glioma but also from pediatric supratentorial glioma in terms of genetic alterations and expression signatures [3-5]. Gliomas arising in the midline of the central nervous system (including the brainstem) commonly harbor the H3.3/3.1-K27M mutation only, while gliomas that arise in the cerebral cortex may contain an alternative H3.3-G34R/V mutation [6-9]. Regional differences in glioma may be a consequence in part of innate differences in the cells from which the tumors arise. Several studies have shown that NSCs from various regions of the brain differ in their capacity to respond to oncogenic stimuli *in vitro* and *in vivo* [18,19]. In addition, human infratentorial and supratentorial low-grade gliomas have distinct expression signatures corresponding to normal astrocytes and NSCs from their respective regions [20], while the expression patterns of G34R/V and K27M mutant gliomas mimic the developmental region in which they arise [8].

One gene found to be expressed at higher levels in infratentorial compared to supratentorial low-grade glioma is paired box 3 (*PAX3*) [20]. Pax3 is a developmental transcription factor that displays a regionalized expression pattern in the neural tube, neural crest, and somite of the vertebrate embryo, with expression decreasing as differentiation progresses [21]. Early in embryogenesis, Pax3 is transiently expressed in the posterior and dorsal neural tube [22-24]. This leads to its expression in the developing CNS to be localized to infratentorial structures such as the brainstem and cerebellum, but not to supratentorial domains such as the cerebral cortex (see P4 in situ hybridization for *Pax3* in [25]). Pax3 is not only necessary for the proper specification of these developing tissues [21,26-28], but also for cell survival as homozygous loss of Pax3 in the mouse leads to severe neural crest and neural tube defects, attributable to an induction of p53-dependent apoptosis [29-31].

Ectopic expression of Pax3 is found to be pro-tumorigenic in sarcomas and neural crest-derived tumors such as melanoma and neuroblastoma [21]. However its role in glioma is less well-understood. Pax3 is re-expressed and upregulated in human glioma relative to normal brain tissue with its expression increasing concomitant with World Health Organization Grade and worsening prognosis, irrespective of tumor location [32]. Functionally, Pax3 has been implicated in promoting proliferation and invasion and inhibiting apoptosis of glioma cell lines, and promoting growth of glioma subcutaneous xenografts [33].

In this study, we sought to gain insight into the unique attributes of BSG by comparing it to high-grade glioma of the cerebral cortex (CG). In an expression array comparing mouse BSG and CG induced by PDGF-B overexpression and Ink4a-ARF loss [34], we identified *Pax3* as being a brainstem-specific marker. Pax3 is regionally expressed in the neonatal mouse brain as well, characterizing a subset of Nestin progenitors in the brainstem but not in the cerebral cortex. Ectopic overexpression of Pax3 in brainstem progenitors inhibits apoptosis *in vitro* and enhances PDGF-B-induced brainstem gliomagenesis *in vivo*, in a region-specific manner. In the absence of p53, while Pax3 no longer inhibits apoptosis, it increases proliferation *in vitro* and maintains its pro-tumorigenic role *in vivo*. Importantly, the regional expression pattern of Pax3 is mirrored in human glioma, revealing a subset of BSG with high *PAX3* expression that associates with alterations in *PDGFRA* and cell cycle regulatory genes and is exclusive of *ACVR1* mutations. These data suggest that regional expression of Pax3 contributes to PDGF-B-induced brainstem gliomagenesis and characterizes a subset of human BSG.

## Materials and methods

### Mice

Nestin-Tva (Ntv-a) and Ntv-a;Ink4a-ARF^−/−^ [34] and Ntv-a;p53^fl/fl^ mice [35] have been described. Olig2-eGFP-L10a [36,37] were bred to Ntv-a;Ink4a-ARF^−/−^ mice to generate Ntv-a;Ink4a-ARF^−/−^;Olig2-eGFP-L10a mice. Pax3-floxed mice [38] were bred to Ntv-a;p53^fl/fl^ mice to generate Ntv-a;p53^fl/fl^;Pax3^fl/fl^ (Pax3-KO) and control Ntv-a;p53^fl/fl^;Pax3^+/+^ mice. Nestin-CFPnuc mice have been previously described, and express the cyan fluorescent protein fused to a nuclear localization signal under the control of the regulatory elements of the Nestin gene [39]. For genotyping, gDNA was isolated from mice using the REDExtract-N-Amp Tissue PCR Kit (Sigma) per the manufacturer’s protocol, using previously published primers. All work with mice was done in accordance with the Duke University Animal Care and Use Committee and the Guide for the Care and Use of Laboratory Animals.

### RCAS/Tv-a glioma mouse modeling

In the RCAS/Tv-a glioma mouse modeling system, avian retroviruses (produced from RCAS plasmids) infect mouse cells expressing Tv-a (the receptor for RCAS viruses). DF1 cells (ATCC) were cultured and transfected with RCAS plasmids as described [35] using Fugene 6 or X-TremeGENE 9 (Roche). RCAS-Y, RCAS-PDGF-B, and RCAS-Cre plasmids are described [40]. RCAS-Pax3 plasmid was obtained from Andrew Bendall (University of Guelph, Ontario) [41]. RCAS-H3.3-K27M plasmid was generated by the C. D. Allis lab (The Rockefeller University, New York) [42]. Generation of PDGF-B-induced brainstem glioma [34,35] and cerebral cortex glioma [43] were as described. Briefly, 1×10^5^ DF1 cells transfected with RCAS plasmids and producing RCAS viruses, were injected into the brainstem or cerebral cortex of neonatal Ntv-a pups (postnatal day 2–4) in 1 μL volume using a custom Hamilton syringe. Combinations of viruses were injected at a 1:1 or a 1:1:1 ratio, and RCAS-Y transfected DF1 cells were used as a negative control. Injected mice were monitored daily and euthanized with CO_2_ upon appearance of signs of brain tumors (enlarged head, ataxia, weight loss up to 25%) or at 12 weeks post-injection in the absence of symptoms.

### FACS of Olig2-eGFP-L10a glioma

Brainstem or Cerebral Cortex Gliomas from Ntv-a;Ink4aARF^−/−^;Olig2-eGFP-L10a mice injected with RCAS-PDGF-B were harvested and dissociated into a single cell suspension as described [35]. Briefly, tissue was digested in papain (Worthington) and DNAse (Sigma Aldrich) at 37°C, followed by three cycles of triturations in ovomucoid (Worthington) and centrifugation at 600 rpm. The resulting cells were passed through a 70 μm strainer, an aliquot of cells was set aside for the unsorted control, and the remainder of cells were sorted using a FACSVantage sorter (BD Bioscience) by the Duke Cancer Institute Flow Cytometry Core into GFP + and GFP- populations. Cell populations were centrifuged, washed in PBS, snap frozen and stored at −80°C for mRNA isolation.

### Immunofluorescence

Ntv-a or Nestin-CFPnuc pups were sacrificed at P3 and their whole brains were fixed in 4% PFA in PBS for 24 hours, cryopreserved in 30% sucrose in PBS for 24–48 hours, followed by embedding in OCT on dry ice/ethanol. Blocks were sectioned using either a Shandon or Leica Cryostat into 12 μm thick sections in a sagittal orientation. Sections were rehydrated in PBS-T (0.1% Triton-X100) and blocked in PBS-T with 10% normal goat serum. Primary antibodies were diluted in PBS-T with 1% BSA overnight at 4 degrees, and secondary antibodies were diluted in PBS-T for 1 hour. Antibodies used were anti-PAX3 (DSHB, concentrated form, 1:200), and anti-GFP (Invitrogen, 1:200); AlexaFluor-488 and −594 secondary antibodies (Invitrogen) were used at 1:200. Slides were mounted with Vectashield with Dapi (Vector Laboratories) and imaged using a Zeiss Axio Imager.

In order to quantify PAX3 and Nestin expression, three Nestin-CFPnuc P3 mice were analyzed. High-powered (20x) pictures of the PAX3-expressing cells in each brainstem region (midbrain, dorsal pons, mid pons, and ventral pons) were taken, 1–3 images per region per section, until at least 6 sections were represented for each region. All pictures were taken using a Zeiss Axio Imager and consistent camera settings. For the cerebral cortex, 2 random 20x images were taken in at least 6 sections per mouse. Using Metamorph Premier software, a threshold was set for positive Nestin and PAX3 staining in each image. For each marker, the same threshold was applied to each image across the dorsal, mid, and ventral pons regions for each section. For the midbrain region, the threshold for PAX3 was consistently lowered slightly to accommodate a lower intensity of the PAX3 staining compared to the other regions. The threshold for Nestin staining in the cerebral cortex was set separately, and remained the same for all sections and all mice. There was no positive nuclear PAX3 staining in any cerebral cortex section. The positive nuclei (size > 200) for each marker were then counted using Integrated Morphometry Analysis. A color threshold was then applied to each Nestin/PAX3 overlay (the same threshold was used across regions for each section), and the number of double-positive cells were counted as described above. The cell counts for each region per section were totaled, and the percentage of Nestin + cells that were double positive per region was calculated. The percentages were averaged together across sections of the same mouse to generate a mean percentage for each region per mouse.

### Mouse and human expression analysis

Frozen BSG and CG samples, along with brainstem and cerebral cortex from age-matched non tumor-bearing mice of the same genetic background (n = 3 for all groups), were sent to Expression Analysis (EA, Durham, NC) for total mRNA isolation and hybridization to Affymetrix GeneChip Mouse Genome 430 2.0 Arrays. Total mRNA samples isolated from Olig2-eGFP-L10a-positive PDGF-B;Ink4aARF^−/−^ glioma cells were sent to EA for hybridization to the same arrays. Data was analyzed using Partek Genomics Suite (Partek Incorporated) for quality control, Robust Multi-Array (RMA) was used for normalization, and differentially expressed genes were determined by one-way ANOVA using the cutoffs described in the text.

Human BSG and CG samples with expression data available from St. Jude [4,44] were selected for analysis. Log-scale Robust Multiarray Analysis (RMA) from the *affy* [45] package in Bioconductor [46] was used for normalization to eliminate systematic differences across the arrays. Differentially expressed genes between brainstem and cerebral cortex glioma samples were identified using the *limma* [47] package in Bioconductor, which utilizes an empirical Bayes’ approach for parameter estimation and significance testing. The False Discovery Rate (FDR) method was employed to control for multiple hypothesis testing. To compare gene expression differences within the brainstem samples, RMA normalization was applied to the arrays where they were then classified based on their normalized PAX3 expression levels (probe 231666_at). Arrays where PAX3 had a normalized expression value > 4 were classified as *PAX3*-High, while all others were classified as *PAX3*-Low. Identification of genes differentially expressed between the two groups was calculated using *limma* as described above. These same classifications of *PAX3*-High and *PAX3*-Low were used for associations with genetic alterations, where the genetic alterations were acquired from previously published data [4,9,11,48].

Human BSG and CG samples with expression data available from Necker-Sick Children [5] were selected for an independent analysis. The Agilent microarray data was processed and normalized using the *limma* [47] package in Bioconductor [46]. The normexp method (normal + exponential convolution model) was used to correct for background intensities. Quantile normalization was used to eliminate systematic differences across all of the arrays. Differentially expressed genes between brainstem and cerebral cortex glioma samples were determined using the methods described above for the first dataset. To compare gene expression differences within the brainstem samples, arrays were classified based on their PAX3 expression levels (probe name: A_23_P502706). Arrays with a PAX3 expression level > 7 were classified as *PAX3*-High, whereas all others were classified as *PAX3*-Low. Identification of genes differentially expressed between the two groups was calculated using *limma* as described above.

### *In vitro* infection of brainstem progenitors with RCAS viruses

Normal brainstem was isolated from Ntv-a or Ntva;p53^fl/fl^ postnatal day 3 (P3) pups and was digested and dissociated as described above for Olig2-eGFP-L10a tumors. Cells were cultured in DMEM supplemented with 10% FBS, 2 mM L-glutamine, 100 units/mL penicillin and 100 μg/mL streptomycin at 37°C and 5% CO_2_, and passaged a maximum of 3 times for experiments. Cells were plated in clear 6-well plates for Annexin V assays, clear 96-well plates for BrdU assays, and white-walled, clear-bottomed 96-well plates for Caspase 3/7 assays.

To concentrate RCAS viruses, DF1 cells transfected with RCAS plasmids were passaged a minimum of 6 times from transfection, and then passaged 1:12. After 3 days, virus-containing media was harvested, centrifuged to remove cell debris, filtered through 0.45 μm pores, and concentrated 100-fold using Retro-X Concentrator (Clontech) per the manufacturer’s instructions.

Brainstem progenitors were plated and infected with RCAS viruses at 1:100. Assays were conducted 3–5 days post-infection, or were split when confluent and subsequently plated for assays. For BrdU assays, the Cell Proliferation ELISA, BrdU Colorimetric kit (Roche) was utilized, per the manufacturer’s instructions using a Molecular Devices Versa Max Tunable Microplate reader. Caspase 3/7 activity was measured using the ApoToxGlo Triplex assay (Promega) per the manufacturer’s instructions using a Turner Biosystems Modulus Microplate Reader. To measure the percentage of cells staining positive for Annexin V, the FITC Annexin V Apoptosis Detection Kit I (BD Pharmingen) was utilized per the manufacturer’s instructions. For cell counting experiments, 50,000 infected cells were plated in 6 well plates in duplicate. For each time point, 2 wells of each line were trypsinized and counted with a Sceptor 2.0 Cell Counter (Millipore). All experiments were done a minimum of three times on at least three independent preparations of progenitor cells.

### Tumor grading and immunohistochemistry

Tumor samples fixed in 10% formalin were embedded in paraffin by the Duke Pathology Core and cut into 5 μm thick sections using a Leica RM2235 microtome. H&E staining was performed using standard protocols. Tumor grading was done using the following criteria: Grade II glioma indicated by increased cellular density only; Grade III glioma indicated by the presence of microvascular proliferation; Grade IV glioma indicated by the presence of pseudopalisading necrosis. Immunohistochemistry (IHC) was performed using an automated processor (Discovery XT, Ventana Medical Systems, Inc.). Anti-PCNA antibody (Calbiochem) was used at 1:2000.

### PCR

DNA was isolated from frozen tumors using the DNeasy Blood and Tissue Kit (Qiagen) per the manufacturer’s instructions. Detection of the recombined Pax3-floxed allele was performed using previously published primers [38] and 60°C annealing temperature. For RT-PCR (both conventional and real time), cultured cells or snap frozen tumors were processed for mRNA isolation using the RNeasy Mini kit (QIAGEN) per the manufacturer’s protocol. cDNA was synthesized from total mRNA using SuperScript II and OligodT primer (Invitrogen). RT-PCR primers for PDGF-B, Forward: 5’-AGTGACCACTCGATCCGCTCCT-3’, Reverse: 5’- TTTGGGGCGTTTTGGCTCGCTG-3’, 55°C annealing temperature. PDGF-B primers amplify both mouse and human PDGF-B cDNA. qRT-PCR reactions were set up with iQ SYBR Green Supermix (BioRad) and run on a Bio-Rad iQ5 Multicolor Real-Time PCR Detection System. Primers for *Pax3*, Forward: 5’- TCCATCCGACCTGGTGCCAT-3’, Reverse: 5’- TTCTCCACGTCAGGCGTTG, and *Actin*, Forward: 5’- TATTGGCAACGAGCGGTTCC-3’, Reverse: 5’- GGCATAGAGGTCTTTACGGATGTC-3’; 60°C annealing temperature. Relative gene expression levels were generated using the ΔΔCt method [49] using *Actin* as the reference gene.

### Western blot

Frozen tumors were either grinded with a mortar and pestle on liquid nitrogen followed by lysis in RIPA buffer containing 1x Protease Inhibitor Cocktail (Sigma Aldrich), 10 mM PMSF, 50 mM NaF, 1 mM NaVO_4_, and 1 mM DTT or lysed and homogenized in the same buffer using a Glas-Col Variable Speed-Reversible Homogenizer set to a motor speed of 80 followed by sonication using a Brason Sonifier 250. Cleared and denatured lysates were run on a NuPAGE 4-12% Bis-Tris gradient gel (Invitrogen), and transferred onto nitrocellulose membrane using the iBlot (Invitrogen). Antibodies were diluted in Odyssey Blocking Buffer (Li-Cor) with 0.2% Tween-20. Blots were imaged using the Odyssey (Li-Cor) per the manufacturer’s instructions. Antibodies used were: anti-PAX3 (DSHB, concentrated form, 1:1000) and anti-ACTIN (Santa Cruz Biotechnology, 1:500). Secondary antibodies from Li-Cor were used at 1:10,000 for IRDye800CW or 1:20,000 for IRDye680LT.

### Statistical analysis

Statistics were calculated using GraphPad Prism 5 software. *In vitro* assays were performed a minimum of 3 times using cells from at least 3 independent P3 litters. Data are represented as the mean with SEM, using student’s paired t-test. qRT-PCR for *Pax3* in mouse tissue was analyzed using student’s unpaired t-test. *In vivo* analyses were performed using log-rank test (for survival) and Fisher’s exact test (for tumor penetrance and grade). For analyses of human data, unpaired t-test was used for the age at diagnosis of *PAX3*-Low versus *PAX3*-High tumors, and Fisher’s exact test was used to test for significance of the association between high Pax3 expression and genetic alterations.

## Results

### Mouse brainstem glioma and cerebral cortex glioma have distinct gene expression signatures

**Figure 1:**
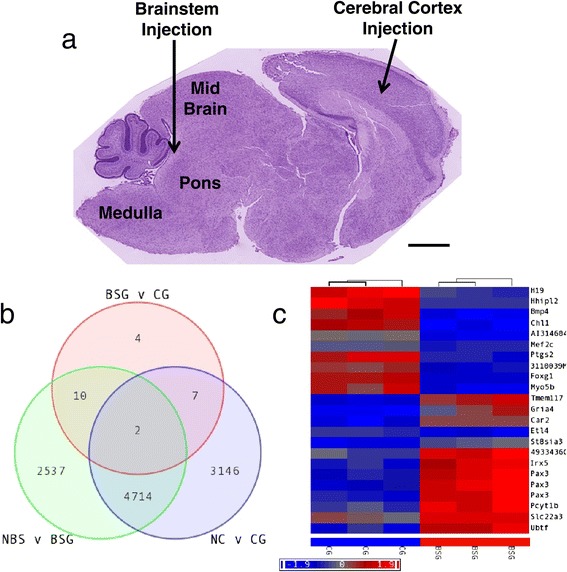
**Regional differences in PDGF**-**B**-**driven mouse glioma. a** Glioma was initiated in the mouse by injecting RCAS-PDGF-B virus into the brainstem or cerebral cortex of neonatal Ntv-a;Ink4a-ARF^−/−^ mice. Shown is a sagittal section of a wildtype postnatal day 4 brain, stained with H&E, indicating the location of brainstem and cerebral cortex injections. Scale bar is 1 mM. **b**-**c** Expression profiling was conducted on the resulting Brainstem Glioma (BSG) and Cerebral Cortex Glioma (CG) and compared with age-matched normal brainstem (NBS) and normal cerebral cortex (NC), n = 3 for each. **b** Venn diagram showing the intersection of genes differentially expressed between NBS and BSG (green circle), between NC and CG (blue circle), and between BSG and CG (red circle). **c** Hierarchical clustering of 23 genes differentially regulated between BSG and CG. FDR-adjusted p-value <0.05 and fold-change ≥2.0.

In order to identify regional differences in glioma, and in particular unique characteristics of those arising in the brainstem, we generated mouse Brainstem Glioma (BSG) and Cerebral Cortex Glioma (CG) by injecting RCAS-PDGF-B virus-producing cells into the brainstem or cerebral cortex, respectively, of postnatal day 2–4 (P2-4) Nestin-Tv-a(Ntv-a);Ink4aARF^−/−^ mice (Figure 1a) [34,43]. The gene expression profiles of the resulting tumors were first compared to their normal tissue counterparts. Using an FDR-adjusted p-value <0.05 and fold-change ≥2.0, 7,869 genes were differentially expressed between normal cortex (NC) and CG (Figure 1b, Additional file 1: Table S1), while 7,263 genes were altered in BSG compared to normal brainstem (NBS) (Figure 1b, Additional file 2: Table S2). Of the latter group, 2,547 genes were specific to the brainstem gliomagenesis process and were not altered during gliomagenesis in the cerebral cortex (Figure 1b). In addition, the comparison between BSG and CG revealed only 23 genes differentially expressed between these two types of tumors (Figure 1b-c, Additional file 3: Table S3), including upregulation of *Irx5* and *Pax3* and downregulation of *Bmp4* and *Foxg1*. We were interested in genes that functionally contribute to the brainstem gliomagenesis process, and so when these 23 genes were intersected with the 2,547 BSG-associated genes, 10 probes, representing 8 brainstem-specific gliomagenesis genes, were identified (Figure 1b, Table 1).

**Table 1:** **8 Genes associated with brainstem gliomagenesis but not cerebral cortex gliomagenesis and differentially expressed between Brainstem Glioma** (**BSG**) **and Cerebral Cortex Glioma** (**CG**) **in the mouse**

**Gene symbol**	**Gene title**	**Fold-** **change BSG vs NBS**	**p**-**value**	**Fold-** **change BSG vs CG**	**p**-**value**
**Pax3**	**paired box gene 3**	4.96	1.04E-05	8.44	1.20E-06
**Pax3**	**paired box gene 3**	3.87	2.19E-04	6.32	2.45E-05
**Irx5**	**Iroquois related homeobox 5** (**Drosophila**)	3.35	2.67E-03	19.01	6.15E-06
**Pax3**	**paired box gene 3**	2.61	2.86E-04	3.70	3.24E-05
Pcyt1b	phosphate cytidylyltransferase 1, choline, beta isoform	2.49	1.11E-05	2.46	1.24E-05
4933436C20Rik	RIKEN cDNA 4933436C20 gene	2.47	4.35E-05	2.99	1.08E-05
Myo5b	myosin VB	−2.41	5.53E-03	−7.59	2.41E-05
Bmp4	bone morphogenetic protein 4	−2.57	3.47E-07	−2.77	1.94E-07
**Chl1**	**cell adhesion molecule with homology to L1CAM**	−7.21	2.66E-06	−12.91	3.66E-07
Al314604	expressed sequence Al314604	−45.25	8.64E-11	−8.89	7.21E-09

Genes in bold are also differentially expressed between Olig2-eGFP-L10a BSG and CG (See Additional file 5: Table S4). NBS = Normal brainstem.

The expression array comparing Brainstem and Cerebral Cortex Gliomas described above was conducted with whole tumor tissue. Therefore it is possible that non-neoplastic cells contributed to some of the expression differences. To investigate this, we utilized the Olig2-eGFP-L10a reporter strain [36,37], in which the eGFP-L10a fusion protein is expressed under the control of the Olig2 promoter. As Olig2 expression marks all tumor cells of our glioma model [34], this effectively labels tumor cells with GFP. Ntv-a;Ink4a-ARF^−/−^;Olig2-eGFP-L10a mice were injected with RCAS-PDGF-B into the brainstem or cerebral cortex to generate BSG or CG, respectively, within 4–6 weeks from injection (Additional file 4: Figure S1a). Tumors were harvested, dissociated, and sorted into GFP-positive and -negative populations. The GFP-positive fractions were analyzed for gene expression differences by microarray, and 118 genes were found differentially regulated between Olig2-BSG and Olig2-CG tumor cells (Additional file 4: Figure S1b, Additional file 5: Table S4). Importantly, of the 8 brainstem-specific gliomagenesis genes in Table 1, the genes highlighted in bold were also differentially regulated between Olig2-BSG and Olig2-CG cells, and presumably play a role in BSG tumor cell biology.

### Pax3 expression characterizes mouse brainstem glioma

**Figure 2:**
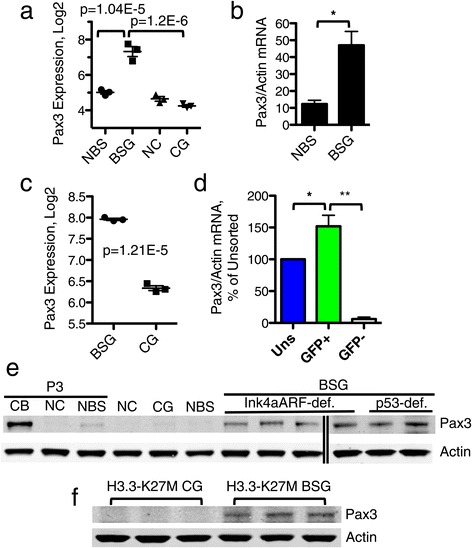
**Pax3 is expressed in mouse Brainstem Glioma. a** Log expression level of *Pax3* for one probe from the expression array in Figure 1. **b** qRT-PCR for *Pax3* in normal brainstem (NBS) and brainstem glioma (BSG) tissue, n = 3 for each. Relative *Pax3* expression is normalized to *Actin*. p = 0.01, unpaired t-test. **c** Log expression level of *Pax3* for one of three probes from the Olig2-eGFP-L10a glioma expression array from Additional file 4: Figure S1b. **d** qRT-PCR for *Pax3* in unsorted (Uns), GFP+, and GFP- fractions of Olig2-eGFP-L10a BSG (n = 5). *Pax3* expression levels are normalized to *Actin* and represented as a percentage of unsorted cells. p = 0.04 Uns vs. GFP+, p = 0.002 GFP+ vs. GFP-, paired t-test. **e** Western blot for PAX3 (53 kDa) in the following mouse tissues from left to right: P3 cerebellum (CB), P3 cerebral cortex (NC), and P3 brainstem (NBS); adult normal cerebral cortex (NC), Cerebral Cortex Glioma (CG), adult normal brainstem (NBS), and Brainstem Glioma (BSG). ACTIN (43 kDa) is shown as a loading control. **f** Western blot for PAX3 (53 kDa) in PDGF-B;H3.3-K27M;p53-deficient Cerebral Cortex Glioma (CG) and Brainstem Glioma (BSG). ACTIN (43 kDa) is shown as a loading control.

Among the 8 brainstem-specific gliomagenesis genes identified in this screen, 3 independent probes for the developmental transcription factor paired box 3 (*Pax3*) showed upregulation in BSG over CG and over normal brainstem (Table 1, Figure 2a). This differential expression pattern was confirmed via qRT-PCR (Figure 2b). *Pax3* was undetectable by qRT-PCR in normal cerebral cortex or CG tissue (n = 3 for each, data not shown). Importantly, *Pax3* was also differentially expressed between Olig2-BSG and -CG cells, with increased expression in BSG relative to CG (Figure 2c, Additional file 5: Table S4). To determine whether *Pax3* expression is confined to the Olig2-tumor cell compartment of BSG, qRT-PCR for *Pax3* was conducted on the sorted GFP-positive and -negative cells, relative to unsorted tumor cells. This revealed that *Pax3* expression is primarily found within the Olig2-tumor cell compartment of BSG (Figure 2d). *Pax3* mRNA was undetectable by qRT-PCR in all compartments of Olig2-eGFP-L10a CG (n = 3, data not shown).

PAX3 protein can also be detected at higher levels in mouse BSG than CG by western blot (Figure 2e). To determine whether high expression of PAX3 occurs in p53-deficient BSG, we injected Ntv-a;p53^fl/fl^ mice with DF1 cells producing RCAS-PDGF-B and RCAS-Cre viruses [35]. In the tumors that arose in these mice, we found PAX3 protein expression at levels comparable to those found in Ink4aARF-deficient tumors (Figure 2e). In addition, to investigate whether the regional expression pattern of Pax3 is relevant in the context of the commonly occurring H3.3-K27M histone mutation, we injected Ntv-a;p53^fl/fl^ mice with DF1 cells producing RCAS-PDGF-B, RCAS-H3.3-K27M, and RCAS-Cre viruses [42]. As shown in Figure 2f, we see high levels of PAX3 protein in PDGF-B;H3.3-K27M;p53-deficient gliomas initiated in the brainstem, but not in the cerebral cortex. Collectively, these data indicate that Pax3 is a brainstem-specific marker of mouse PDGF-B-driven glioma in the context of Ink4aARF-deficiency, p53-deficiency, as well as H3.3-K27M expression.

### Pax3 is regionally expressed in the neonatal mouse brain

**Figure 3:**
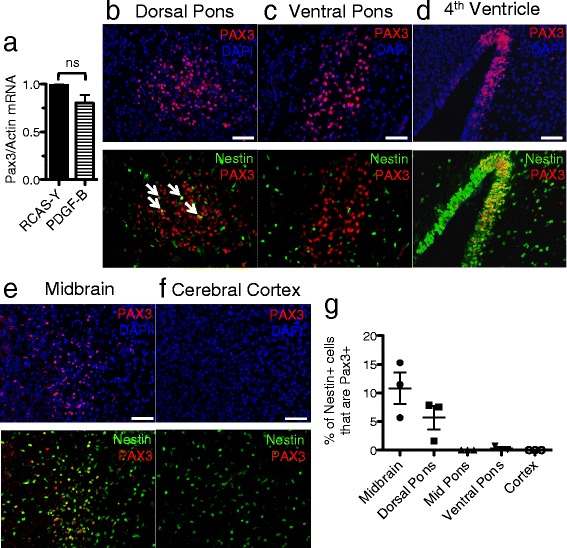
**Regional expression of Pax3 in the neonatal mouse brain. a** qRT-PCR for *Pax3* in RCAS-PDGF-B- versus RCAS-Y-infected brainstem progenitor cells. *Pax3* levels are normalized to *Actin*. **b**-**f** Immunofluorescence for PAX3 (red) and DAPI (blue) (upper panels), and PAX3 (red) and Nestin-CFP (green) (lower panels) in P3 Nestin-CFPnuc brain; 20x magnification, scale bar is 50 μM in **b** dorsal pons (white arrows point to Nestin+/PAX3+ cells), **c** ventral pons, **d** 4^th^ ventricle roof, **e** midbrain, **f** cerebral cortex. **g** The percentage of Nestin+ cells in each of the indicated brain regions that also express PAX3, quantified as described in the Methods section.

High Pax3 expression characterizes PDGF-B-driven mouse BSG while its expression is lower in glioma arising in the cerebral cortex. We speculated that enhanced PDGF signaling induced by PDGF-B ligand might upregulate Pax3 in the brainstem and be responsible for its high expression level. However, when brainstem progenitor cells isolated from Ntv-a P3 mice were infected *in vitro* with RCAS-PDGF-B virus, *Pax3* mRNA levels did not increase (Figure 3a).

As Pax3 is a developmental transcription factor whose expression normally decreases as differentiation proceeds, we next investigated whether Pax3 is expressed at the time of tumor initiation in the brainstem, which in our mouse model is P2-4. By Western blot, PAX3 protein was detected in wildtype P3 brainstem but not cerebral cortex (Figure 2e, cerebellum is shown as a positive control), consistent with its expression pattern in glioma. To further characterize the expression pattern of Pax3 in the developing brain, we conducted immunofluorescence for PAX3 protein in the P3 mouse brain and found several regions of expression within and around the developing brainstem. Distinct clusters of cells immunoreactive for PAX3 were found in the dorsal, mid, and ventral pons (Figure 3b-c, Additional file 6: Figure S2a-b, upper panels). In addition, we found PAX3-positive cells lining the roof of the 4^th^ ventricle and radiating up and out from there into portions of the midbrain, which lies just anterior to the mouse pons (Figure 3d-e, upper panels). PAX3-positive cells were also found occasionally lining the floor of the 4^th^ ventricle (Additional file 6: Figure S2c, upper panels). By contrast, there were no PAX3-positive cells detected by immunofluorescence in the cerebral cortex (Figure 3f, upper panel).

We next investigated whether Pax3 expression characterizes any Nestin-progenitors, the targeted cell-of-origin in our glioma mouse model, in the P3 mouse brainstem. We conducted co-immunofluorescence for cyan fluorescent protein (CFP) and PAX3 in the P3 brain of Nestin-CFPnuc mice [39] which express CFP fused to a nuclear localization signal under the control of the Nestin promoter. PAX3 expression was found in a subset of Nestin progenitors in the dorsal pons, the roof and floor of the 4^th^ ventricle, and the midbrain (Figures 3b, d, e, and Additional file 6: Figure S2c, lower panels, and quantified in Figure 3g). Conversely, we found very little to no co-expression of Nestin and PAX3 in the ventral or mid pons and none in the cerebral cortex (Figure 3c, f, and S2b, lower panels, quantified in Figure 3g). Thus the regional expression pattern of Pax3, and its co-expression with Nestin, in the neonatal mouse brain correlates with its expression in glioma.

### Pax3 inhibits apoptosis of brainstem progenitor cells

**Figure 4:**
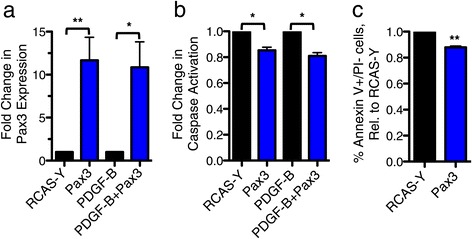
**Pax3 inhibits apoptosis of brainstem progenitor cells.** Brainstem progenitor cells from P3 Ntv-a mice were cultured *in vitro* and infected with the indicated RCAS viruses. **a** qRT-PCR for *Pax3* in the infected progenitor cells, normalized to *Actin*. Levels for Pax3-infected cells are expressed relative to RCAS-Y-infected cells, p = 0.007 paired t-test, and levels for PDGF-B + Pax3-infected cells are expressed relative to PDGF-B-infected cells, p = 0.03 paired t-test. **b** Caspase 3/7 activation of Pax3-infected cells is normalized to that of RCAS-Y-infected cells, p = 0.03 paired t-test, while caspase 3/7 activation of PDGF-B + Pax3-infected cells is normalized to that of PDGF-B-infected cells, p = 0.02 paired t-test. **c** Annexin V staining of RCAS-Pax3- versus RCAS-Y-infected progenitor cells. The percentage of Pax3-infected cells that were AnnexinV+/PI- was normalized to the percentage of RCAS-Y-infected cells that were AnnexinV+/PI-. p = 0.005, paired t-test.

To investigate the functional role of Pax3 in brainstem progenitor cells, the brainstem region (including the midbrain and pons) from P3 Ntv-a mice was isolated, dissociated, and cultured *in vitro*. The Nestin-expressing progenitor cells were infected with RCAS-Y, RCAS-Pax3, RCAS-PDGF-B, or a 1:1 combination of RCAS-Pax3 and RCAS-PDGF-B (Figure 4a and Additional file 7: Figure S3a). RCAS-Pax3 alone, or in combination with PDGF-B, did not directly increase proliferation of brainstem progenitors based on BrdU incorporation (Additional file 7: Figure S3b). However, Pax3 inhibited basal apoptosis as evidenced by caspase 3/7 activation (Figure 4b). Similarly, Pax3 overexpression reduced the percentage of cells staining positive for Annexin V, a marker for early apoptosis, when compared to RCAS-Y-infected cells (Figure 4c).

### Pax3 enhances PDGF-B-induced brainstem gliomagenesis *in vivo*

**Figure 5:**
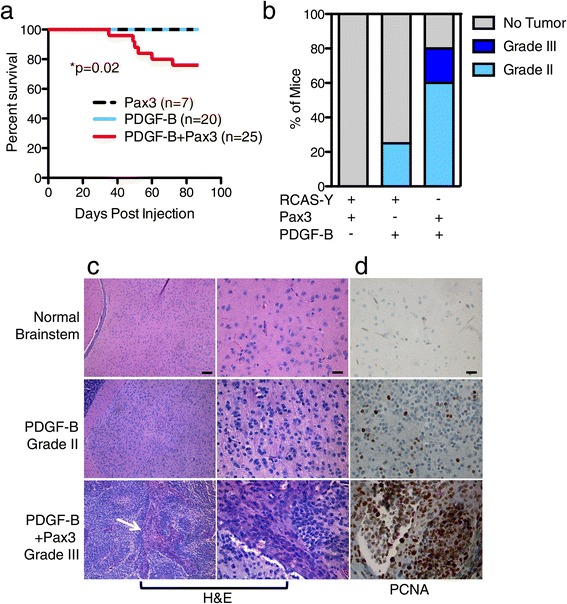
**Pax3 enhances PDGF**-**B**-**induced Brainstem Gliomagenesis. a** Kaplain-Meier survival curve of Ntv-a mice injected with DF1 cells expressing RCAS-Pax3, RCAS-PDGF-B, or RCAS-PDGF-B + RCAS-Pax3. **b** Mice from **(a)** were sacrificed at the onset of tumor symptoms or at 12 weeks in the absence of symptoms. Brains were harvested, fixed in formalin, and analyzed for the presence of tumors using hematoxylin and eosin (H&E) staining, and the tumors graded as described in Materials and Methods. Shown is the percentage of mice in each group with no tumor, grade II glioma, and grade III glioma. **c**-**d** Representative H&E staining **(c)** and IHC for proliferating cell nuclear antigen, PCNA **(d)** of normal brainstem (top row), PDGF-B-induced grade II BSG (middle row), and PDGF-B + Pax3-induced grade III BSG (bottom row). **c** Magnification is 10x in the left panels and 40x in the right panels. White arrow in lower-left panel indicates microvascular proliferation, which is enlarged in the 40x panel to its right. Scale bar for 10x is 100 μM, 40x is 25 μM. **d** 40x magnification; Scale bar is 25 μM.

In order to determine if Pax3-induced inhibition of apoptosis is sufficient to induce glioma *in vivo*, P3 Ntv-a mice were injected in the brainstem with RCAS-Pax3, RCAS-PDGF-B, or a 1:1 mixture of both viruses. While PDGF-B overexpression alone led to asymptomatic low-grade glioma in 5 out of 20 mice, Pax3 overexpression alone did not lead to tumor formation (Figure 5a-c). When compared to PDGF-B injection alone, the addition of Pax3 to PDGF-B significantly reduced survival (Figure 5a) and increased tumor penetrance to 20 out of 25 mice (p = 0.0003, Figure 5b). Importantly, addition of Pax3 to PDGF-B overexpression caused a subset of tumors to progress to high-grade glioma based on the presence of microvascular proliferation (0/5 PDGF-B versus 5/20 PDGF-B + Pax3 high-grade, p = 0.056, Figure 5b-c). The Pax3-induced high-grade gliomas harbored increased proliferation compared to the low-grade tumors induced by PDGF-B alone based on PCNA staining (Figure 5d). The pro-tumorigenic role of Pax3 in glioma is brainstem-specific, as co-injection into the cortex of both PDGF-B and Pax3 did not significantly reduce survival or increase tumor penetrance (p = 1.0) or grade (p = 1.0) compared to injection of PDGF-B alone (Additional file 8: Figure S4). Therefore, in the presence of enhanced PDGF-B signaling, Pax3 is sufficient to generate aggressive high-grade BSG, a feat that PDGF-B alone cannot achieve in the brainstem.

### Pax3 promotes proliferation and gliomagenesis in the brainstem independent of p53

**Figure 6:**
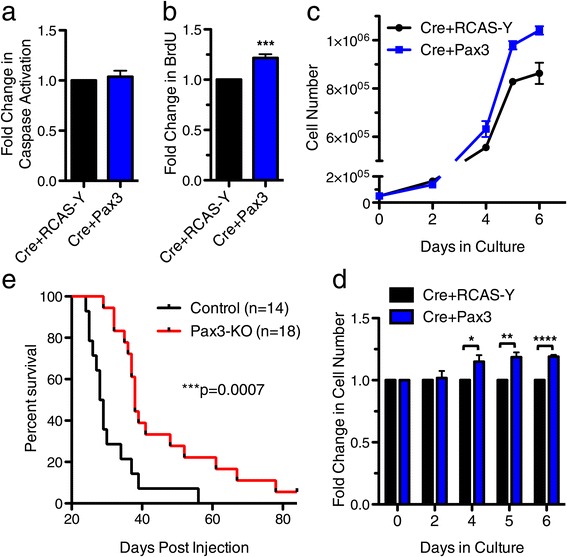
**Pax3 promotes proliferation** ***in vitro*** **and brainstem gliomagenesis** ***in vivo*** **in the absence of p53. a**-**d** Brainstem progenitor cells from P3 Ntv-a;p53^fl/fl^ mice were cultured *in vitro*, infected with the indicated RCAS viruses, and assayed for **(a)** Caspase 3/7 activity at 72 hours after plating, normalized to Cre + RCAS-Y, **(b)** BrdU incorporation at 72 hours after plating, normalized to Cre + RCAS-Y, p = 0.0007 paired t-test, and **(c-d)** cell number at the indicated time points after plating. **c** One representative experiment conducted in one progenitor line. **d** The fold change in Cre + Pax3 cells over Cre + RCAS-Y cells at each time point compiled from multiple progenitor lines, p = 0.03 (day 4), p = 0.005 (day 5), p < 0.0001 (day 6), paired t-test. **e** Kaplan-Meier survival curve of Ntv-a;p53^fl/fl^ mice (Control) and Ntv-a;p53^fl/fl^;Pax3^fl/fl^ mice (Pax3-KO) injected with RCAS-PDGF-B + RCAS-Cre into the brainstem at P2-4.

As the majority of human BSG are p53-deficient, we investigated the role of Pax3 in our BSG mouse model in the absence of this tumor suppressor. Brainstem progenitor cells were isolated from P3 Ntv-a;p53^fl/fl^ mice and infected with RCAS-Cre to knock-out p53 along with either RCAS-Y or RCAS-Pax3. In the absence of p53, Pax3 overexpression no longer inhibited caspase 3/7 activation (Figure 6a). However, in these p53-deficient progenitor cells, Pax3 overexpression did lead to a significant increase in cell proliferation, indicated by BrdU incorporation (Figure 6b) and cell counting (Figure 6c-d).

In order to test whether Pax3 is functionally required for the initiation or progression of p53-deficient BSG *in vivo*, we crossed conditional Pax3 knockout mice [38] into our p53-deficient BSG model [35]. Neonatal Ntv-a;p53^fl/fl^;Pax3^fl/fl^ mice (Pax3-KO) and Ntv-a;p53^fl/fl^;Pax3^+/+^ mice (Control), were injected with RCAS-PDGF-B and RCAS-Cre. Although both groups of mice succumbed to high-grade BSG, the Pax3-KO mice displayed a significant 33% increase in survival (median survival 38 days versus 28.5 days, Figure 6e). Recombination of the Pax3-floxed allele was confirmed via PCR of gDNA and loss of PAX3 protein expression was confirmed via Western blot in Pax3-KO tumors (Additional file 9: Figure S5a-b). The increased survival of Pax3-KO mice suggests that Pax3 functionally contributes to brainstem gliomagenesis, although it is ultimately not required as tumors can quickly evade the loss of Pax3.

### High Pax3 expression defines a novel subset of human BSG

**Figure 7:**
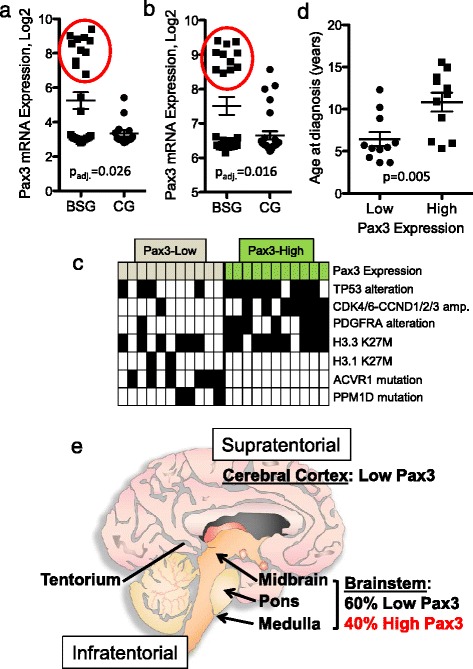
**High Pax3 expression characterizes a subset of human Brainstem Glioma. a**-**b** Previously published expression profiles of human BSG and CG samples: post-treatment autopsy samples from St. Jude **(a)** and pre-treatment biopsy samples from Necker-Sick Children **(b)** were analyzed for gene expression differences as described in Materials and Methods. Log expression level of *PAX3* in BSG versus CG. Red circles indicate the 11/28 **(a)** and 11/25 **(b)** of BSG samples with high *PAX3* expression. **c** Table showing *PAX3*-High and *PAX3*-Low tumors from the dataset in **(a)** and their genetic alterations. Black squares indicate a sample is positive for that alteration. *PDGFRA* alteration includes *PDGFRA* gain, detected either by SNP array or FISH, or mutation (single nucleotide variants, indels, and structural variants), detected by whole-genome sequencing. **d** Age at diagnosis of *PAX3*-Low versus *PAX3*-High human BSG. **e** Schematic of the human brain showing the regional differences in Pax3 expression in glioma.

To correlate our observations regarding Pax3 expression and function in mouse glioma with human disease, pediatric BSG (all of which were characterized as DIPG) and CG samples were utilized from previously published gene expression profiles, one comprised of post-treatment autopsies [4,44], and one comprised of pre-treatment biopsies [5]. The BSG and CG profiles from each dataset were compared, generating lists of significantly differentially expressed genes (FDR-adjusted p-value <0.05, Additional file 10: Table S5 and Additional file 11: Table S6). *PAX3* was upregulated in BSG relative to CG in both datasets, 3.7-fold in the autopsy samples (Figure 7a), and 1.8-fold in the biopsy samples (Figure 7b).

Interestingly, high *PAX3* expression characterized approximately 40% of the BSG samples in both datasets (11/28, Figure 7a, and 11/25, Figure 7b). To glean information regarding the tumors with high *PAX3* expression, we first compared gene expression profiles of the *PAX3*-Low versus *PAX3*-High BSG samples from both datasets. Besides *PAX3*, there were very few genes that met the criteria for significance (adjusted p-value < 0.05, Additional file 12: Table S7 and Additional file 13: Table S8), and none that shed any additional light on the biology of the *PAX3*-High subset. We next compared the genetic alterations occurring in *PAX3*-Low versus *PAX3*-High BSG samples from Figure 7a [4,9,11,48]. As is shown in Figure 7c, Table 2, and Additional file 14: Table S9, high *PAX3* expression significantly associated with wildtype *ACVR1*, *PDGFRA* amplification or mutation, and *CDK4*/*6*-*CCND1*/*2*/*3* amplification. In addition, *PPM1D* and H3.1-K27M mutations were found exclusively in *PAX3*-Low tumors (27% and 18%, respectively), although these relationships were not significant due to their low frequency. The majority of *PAX3*-High tumors also contained *TP53* alterations (82%) and the H3.3-K27M mutation (82%). Lastly, compared to children with *PAX3*-Low BSG, those harboring *PAX3*-High BSG were significantly older at diagnosis (Figure 7d). Collectively, these data describe a novel subset of human BSG with high Pax3 expression that are commonly characterized by increased PDGF signaling, and highlight an important regional difference between pediatric gliomas arising in the cerebral cortex (supratentorial) and the brainstem (infratentorial) (Figure 7e).

**Table 2:** **Genetic alterations associated with Pax3 expression in human Brainstem Glioma**

**Genetic alteration**	**Pax3-** **low**	**Pax3-** **high**	**P-** **value** ^**b**^
**ACVR1 mutation**	6/11	0/11	*0.01
**PDGFRA alteration** ^**a**^	1/11	7/11	*0.02
**CDK4**/**6**-**CCND1**/**2**/**3 amp**	1/11	7/11	*0.02
**TP53 alteration**	4/11	9/11	0.08
**PPM1D mutation**	3/11	0/11	0.2
**Histone H3.3**-**K27M**	6/11	9/11	0.4
**Histone H3.1**-**K27M**	2/11	0/11	0.5

^a^Includes PDGFRA gain, detected either by SNP array or FISH, or mutation (single nucleotide variants, indels, and structural variants), detected by whole-genome sequencing.

^b^Fisher’s Exact Test.

*p-value < 0.05.

## Discussion

Brainstem Glioma (BSG) is a distinct disease, biologically and clinically, from glioma of the cerebral cortex (CG). While decades of research has provided insight into the cellular, molecular, and genetic alterations occurring in CG and guided therapeutic strategies for that disease, the field of BSG research is still in its infancy. In an effort to identify unique characteristics of BSG, we compared gene expression profiles of mouse PDGF-B-induced BSG and CG, and identified 23 genes that are differentially regulated between gliomas arising in the two regions. Among those genes, 8 were associated with the gliomagenesis process in the brainstem but not in the cerebral cortex—these genes we identify as “brainstem-specific gliomagenesis genes”. Of these genes, *Pax3*, *Irx5*, and *Chl1* were also differentially regulated between the Olig2-compartments of BSG and CG suggesting that they represent biological differences within the tumor cells. As Olig2 characterizes the majority of human BSG, particularly the oligodendroglial (PDGFRA) and H3-K27M subgroups [5,8,50,51], as well as marks a candidate cell-of-origin for BSG [52], the expression profile of these cells has important implications for at least a subset of the human disease.

Due to the controlled nature of this experiment, the gene expression signatures identified in this study can be primarily attributed to regional characteristics of the brainstem and cerebral cortex. It has not been previously reported that gliomas induced with the same genetic alterations in different regions of the mouse brain have distinct gene expression. We have found mouse BSG to harbor a distinct expression pattern of developmental homeobox genes and transcription factors, including *Pax3*, *Irx5*, *Foxg1*, and *Lhx2*. Overall, these observations are similar to those made by others in comparing human infratentorial to supratentorial low-grade glioma [20], and BSG to other pediatric high-grade glioma [5]. The latter study did not include *PAX3* in their list of genes upregulated in BSG, however, potentially due to the inclusion of thalamic gliomas in their high-grade glioma group, some of which could harbor high *PAX3* expression.

Herein is the first report of Pax3 expression in BSG. Importantly, we find regional expression of Pax3 in mouse PDGF-B-driven glioma in the context of Ink4aARF-loss, p53-loss, and overexpression of H3.3-K27M that is mirrored in human glioma. *PAX3* mRNA is expressed at higher levels in BSG compared to CG in two independent sets of human samples, which include both pre-treatment biopsies and post-treatment autopsies. The analyses also revealed that approximately 40% of human BSG harbor relatively high *PAX3* expression and that these tumors associate with increased PDGFRA signaling and alterations of cell-cycle regulatory genes, both of which characterize our PDGF-B-driven BSG mouse model. The majority of *PAX3*-High tumors also contain *TP53* alterations and the H3.3-K27M mutation, genetic alterations that also coexist with Pax3 expression in our mouse model. Collectively, these data suggest that *PAX3*-High tumors may be part of the oligodendroglial and the H3-K27M subgroups previously described to harbor *PDGFRA* alterations [5,10]. In addition, *PPM1D*, H3.1-K27M, and *ACVR1* mutations occur only in *PAX3*-Low tumors. *ACVR1* mutations have also been reported to be mutually exclusive of *PDGFRA* alterations [11,13], although as we did not observe a link between PDGF signaling and *PAX3* expression, we believe the exclusivity between high *PAX3* and *ACVR1* mutation to be independent of *PDGFRA*. Consistent with *ACVR1* mutations occurring in younger patients [11], Pax3-High (and thus *ACVR1* wildtype) tumors are associated with older patients in this study.

The regional expression pattern of Pax3 in glioma correlates with its expression in the developing mouse brain, pointing to the possibility that the expression level of Pax3 in different regions of the developing brain dictate its expression level in gliomas arising in those regions. It is plausible that Pax3 is highly expressed in BSG because the gliomagenesis process prevents its normal downregulation, although this has not been directly tested. In support of this, however, several populations of Pax3-positive progenitor cells exist throughout the P3 mouse brainstem. A subset of these cells co-express the stem/progenitor cell marker Nestin, particularly in the dorsal pons and midbrain, locations in the mouse in which Pax3-High BSG are localized. As Nestin cells are the targeted cell-of-origin for the BSG model used here, it is possible that Nestin+/Pax3+ progenitors give rise to Pax3-High glioma in this mouse model. Although a Nestin+ cell is a candidate cell-of-origin for human glioma [53], the cell type(s) and the location of the cell(s) giving rise to human BSG specifically are still in question. The majority of human BSG are at diagnosis localized primarily to the ventral pons, suggesting a potential ventral location for the cell-of-origin [52]. This would be in contradiction to a dorsal Nestin+/Pax3+ cell-of-origin as discussed here, and represents a limitation of this model. However we do not yet know the identity or location of any Nestin+/Pax3+ progenitors in the developing human pons, and as there are significant differences between mouse and human brainstem anatomy, we cannot be certain that the dorsal Nestin+/Pax3+ progenitors identified here in the mouse correlate with a dorsal location in the human pons. Further investigation into the expression of Pax3 along with other glioma markers in the developing mouse and human brainstem will be the focus of future studies to hopefully clarify this issue.

Investigation into the function of Pax3 in the brainstem revealed that ectopically expressed Pax3 is not oncogenic in the absence of any other genetic alterations. While *in vitro*, Pax3 overexpression inhibits basal apoptosis, this effect is insufficient to induce tumor formation *in vivo*. When combined with the pro-proliferative effects of increased PDGF signaling, a common event found in *PAX3*-High human BSG, ectopic Pax3 enhances gliomagenesis, increasing tumor frequency and promoting progression to a high-grade malignancy. Although we are unable to determine the percentage of tumor cells expressing both ectopic PDGF-B and Pax3 in this model, the survival advantage associated with both seen here suggests that the tumorigenesis process would select for doubly infected cells.

Pax3-induced inhibition of apoptosis is seemingly depen-dent on p53, as deletion of p53 in brainstem progenitors *in vitro* abolishes the anti-apoptotic effects of Pax3. This is in accordance with literature describing a direct interaction between Pax3 and p53 proteins leading to degradation of p53 [30,54]. Interestingly, in the absence of p53, Pax3 promotes the proliferation of brainstem progenitors, which may explain how deletion of Pax3 in p53-deficient BSG delays gliomagenesis *in vivo*. These pro-survival effects of Pax3 suggest that it can cooperate with other oncogenic drivers, either through inhibition of p53-dependent apoptosis or by promoting proliferation, functions that it provides to other types of cancer cells, including glioblastoma cell lines [33,55-57]. It should be noted that the *in vitro* experiments described here were conducted in the presence of serum, which induces differentiation. As Pax3 function is most likely dependent upon differentiation status, studying the effects of Pax3 in the context of stem cell conditions should be a focus of future research.

Collectively, our functional data suggest that Pax3 is able to promote the initiation as well as the progression of brainstem gliomagenesis when combined with other genetic alterations. As Pax3 has been found expressed in cerebral cortex glioma [32], infratentorial low-grade glioma [20], as well as high grade BSG as shown here, it appears that Pax3 is expressed in glioma (albeit at different levels) regardless of the tumor grade or location, and may play a functional role in all scenarios dependent on the context of additional genetic alterations. We find here that Pax3 increases the frequency of both low- and high-grade BSG in the context of PDGF signaling, supporting its role as an oncogene in both low- and high-grade brainstem lesions. Although results herein show a lack of Pax3 upregulation and function in cerebral cortex gliomas of mice, Chen et al. showed that Pax3 levels correlate with increasing WHO grade of supratentorial gliomas [32] and subsequently Xia et al. showed that Pax3 functionally promotes survival of GBM cell lines and xenografts originally isolated from supratentorial tumors [33]. Based on our direct comparison between BSG and CG, we would expect the levels of Pax3 expression in these supratentorial gliomas to be lower than in BSG. The functional discrepancies could be explained by varying contexts of genetic alterations, differences between mouse and human tumor biology, or an inhibitory effect on Pax3 expression or function of the cerebral cortex stroma in mice.

In summary, the data presented here show that mouse BSG and a subset of human BSG highly express Pax3, distinguishing these tumors from gliomas arising in the cerebral cortex. This expression pattern of Pax3 is mirrored in the developing brain, which may account for the gene’s regional specificity in glioma. We have identified a subset of human BSG with high *PAX3* expression that associates with *PDGFRA* alterations and amplification of cell-cycle regulatory genes, and is exclusive of *ACVR1* mutations. Functional studies show that in addition to being a regional marker for BSG, Pax3 also inhibits apoptosis and promotes proliferation of brainstem progenitor cells, and contributes to PDGF-B-induced brainstem gliomagenesis *in vivo*. Important complements to these studies in the future will be to investigate the function of Pax3 in human BSG cells, both in cell lines and xenografts, as well as in epigenetically-induced models to study how the presence of the H3.3-K27M mutation contributes to or alters Pax3 function. Further investigation into the mechanisms upstream and downstream of Pax3 will increase our understanding of this subset of BSG and may lead to the identification of targets or pathways that could be exploited for therapeutic purposes.

## Additional files

Additional file 1: Table S1. Differentially expressed genes between mouse normal cortex (NC) and Cerebral Cortex Glioma (CG), FDR-adjusted p-value < 0.05, fold-change ≥ 2. Additional file 2: Table S2. Differentially expressed genes between mouse normal brainstem (NBS) and Brainstem Glioma (BSG), FDR-adjusted p-value < 0.05, fold-change ≥ 2. Additional file 3: Table S3. Differentially expressed genes between mouse Brainstem Glioma (BSG) and Cerebral Cortex Glioma (CG), FDR-adjusted p-value < 0.05, fold-change ≥ 2. Additional file 4: Figure S1. Regional differences in Olig2-Glioma cell compartments. a Kaplan-Meier survival curve of Ntv-a;Ink4a-ARF^−/−^;Olig2-eGFP-L10a mice injected with RCAS-PDGF-B into the brainstem or cerebral cortex at P2-4 to generate Brainstem Glioma (BSG) and Cerebral Cortex Glioma (CG), respectively. b Gliomas from (a) were harvested, dissociated, and sorted into GFP + and GFP- compartments by FACS and the GFP + BSG and CG samples (n = 3 for each) were compared using expression profiling. Shown is hierarchical clustering of 118 genes differentially regulated between BSG and CG Olig2-cells. p <0.01 and fold change ≥2.0. Additional file 5: Table S4. Differentially expressed genes between Olig2-eGFP-L10a gliomas initiated in the brainstem (BS) and the cerebral cortex (CX), p-value < 0.01 and fold change ≥2.0. Additional file 6: Figure S2. Regional expression of Pax3 in the neonatal mouse brain. a Immunofluorescence for PAX3 (red) in P3 Ntv-a pons. Nuclei are stained with DAPI (blue). 10x magnification, scale bar is 100 μM. White boxes indicate dorsal, mid, and ventral pons populations of PAX3-expressing cells. 4^th^ ventricle and Cerebellum (Cb) are indicated for reference. b-c Immunofluorescence for PAX3 (red) and DAPI (blue), upper panels; PAX3 (red) and Nestin-CFP (green), lower panels. 20x magnification, scale bar is 50 μM. b mid pons, c 4^th^ ventricle floor: left panels are representative of the majority of sections analyzed in which the Nestin-progenitors lining the ventricle are negative for PAX3. Right panels are representative of rare sections in which a subset of the Nestin-progenitors lining the ventricle expresses PAX3. Additional file 7: Figure S3. Normal brainstem progenitors isolated from P3 Ntv-a mice were infected with RCAS-Y, RCAS-Pax3, RCAS-PDGF-B, or RCAS-Pax3 + RCAS-PDGF-B. a mRNA from infected cells was isolated and analyzed for PDGF-B expression by RT-PCR. PCR primers for PDGF-B amplify endogenous mouse PDGF-B as well as the human RCAS-PDGF-B. b BrdU incorporation of normal brainstem progenitors from Ntv-a P3 mice infected with the RCAS viruses as indicated below the graph (RCAS-Y vector was used to control for the total amount of virus). Data is represented as fold-change over RCAS-Y. Additional file 8: Figure S4. Ntv-a mice were injected into the cerebral cortex with DF1 cells expressing RCAS-Pax3, RCAS-PDGF-B, or RCAS-PDGF-B + RCAS-Pax3 on P2-4, and monitored for signs and symptoms of brain tumors. a Kaplain-Meier survival curve; p = 0.5, PDGF-B vs. PDGF-B + Pax3. b Mice from the experiment in (a) were sacrificed at the onset of tumor symptoms, or at 12 weeks in the absence of symptoms, and the brains of all mice were analyzed for the presence of glioma using hematoxylin and eosin (H&E) staining, and their tumors graded as described in Materials and Methods. Shown is the percentage of mice in each group with no tumor, grade II, III, and IV glioma. Additional file 9: Figure S5. Deletion of Pax3 in p53-deficient Brainstem Glioma. Ntv-a;p53^fl/fl^ mice (Control) and Ntv-a;p53^fl/fl^;Pax3^fl/fl^ mice (Pax3-KO) were injected with RCAS-PDGF-B + RCAS-Cre into the brainstem at P2-4. Representative Control and Pax3-KO tumors were analyzed for recombination of the Pax3-floxed allele by PCR of gDNA (a) and for PAX3 protein expression by Western blot (b). Additional file 10: Table S5. Differentially expressed genes between human Brainstem Glioma (Brainstem) and Cerebral Cortex Glioma (Cortical) from post-treatment autopsy samples, FDR-adjusted p-value < 0.05. Additional file 11: Table S6. Differentially expressed genes between human Brainstem Glioma (Brainstem) and Cerebral Cortex Glioma (Cortical) from pre-treatment biopsy samples, FDR-adjusted p-value < 0.05. Additional file 12: Table S7. Differentially expressed genes between *PAX3*-High and *PAX3*-Low Brainstem Glioma post-treatment autopsy samples, FDR-adjusted p-value < 0.05. Additional file 13: Table S8. Differentially expressed genes between *PAX3*-High and *PAX3*-Low Brainstem Glioma pre-treatment biopsy samples, FDR-adjusted p-value < 0.05. Additional file 14: Table S9. Genetic alterations associated with Pax3 expression in human Brainstem Glioma samples.
