# Supplementary figures and images for: Pax3 expression enhances PDGF-B-induced brainstem gliomagenesis and characterizes a subset of brainstem glioma
Source: Acta Neuropathol Commun. 2014 Oct 21;2:134. doi: 10.1186/s40478-014-0134-6 (PMC4210596; doi:10.1186/s40478-014-0134-6)

Figure S1

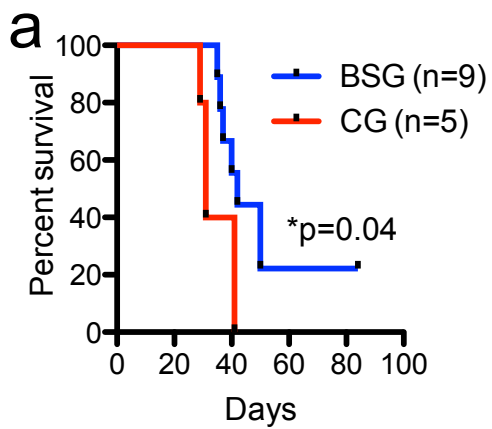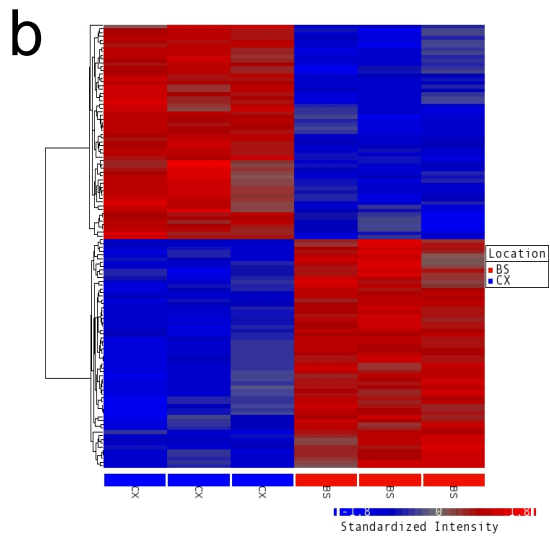

Supplement: Additional file 4: Figure S1. — Regional differences in Olig2-Glioma cell compartments. a Kaplan-Meier survival curve of Ntv-a;Ink4a-ARF−/−;Olig2-eGFP-L10a mice injected with RCAS-PDGF-B into the brainstem or cerebral cortex at P2-4 to generate Brainstem Glioma (BSG) and Cerebral Cortex Glioma (CG), respectively. b Gliomas from (a) were harvested, dissociated, and sorted into GFP + and GFP- compartments by FACS and the GFP + BSG and CG samples (n = 3 for each) were compared using expression profiling. Shown is hierarchical clustering of 118 genes differentially regulated between BSG and CG Olig2-cells. p <0.01 and fold change ≥2.0. [file 40478_2014_134_MOESM4_ESM.pdf]

Figure S2

**a**

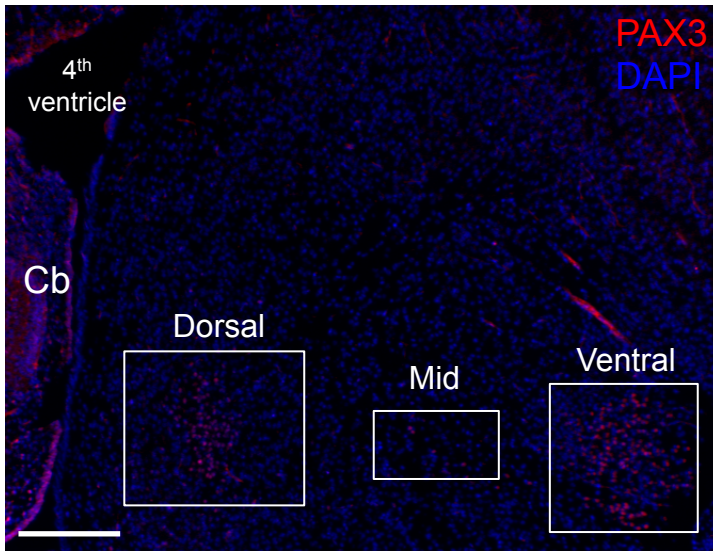

**b**

Mid BS

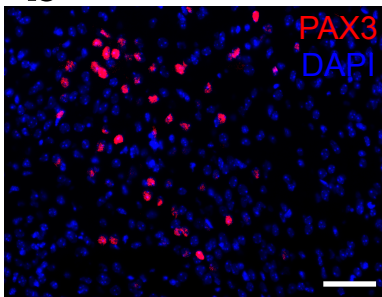

**c**

4<sup>th</sup> Ventricle Floor

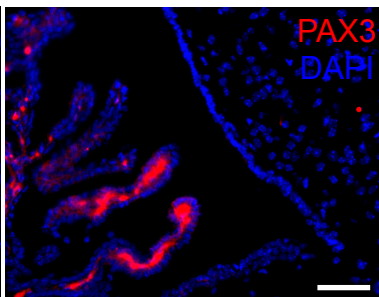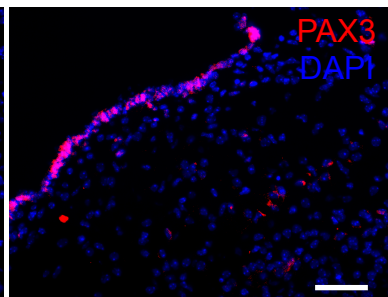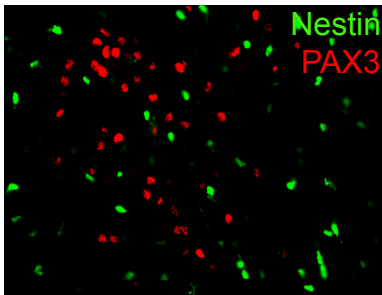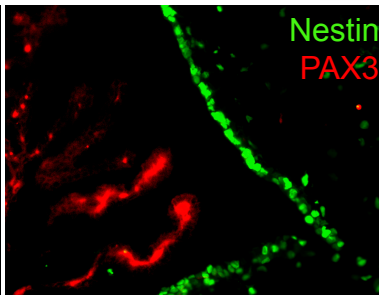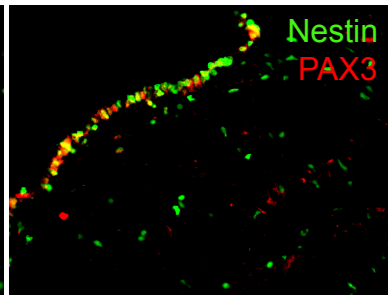

Supplement: Additional file 6: Figure S2. — Regional expression of Pax3 in the neonatal mouse brain. a Immunofluorescence for PAX3 (red) in P3 Ntv-a pons. Nuclei are stained with DAPI (blue). 10x magnification, scale bar is 100 μM. White boxes indicate dorsal, mid, and ventral pons populations of PAX3-expressing cells. 4th ventricle and Cerebellum (Cb) are indicated for reference. b-c Immunofluorescence for PAX3 (red) and DAPI (blue), upper panels; PAX3 (red) and Nestin-CFP (green), lower panels. 20x magnification, scale bar is 50 μM. b mid pons, c 4th ventricle floor: left panels are representative of the majority of sections analyzed in which the Nestin-progenitors lining the ventricle are negative for PAX3. Right panels are representative of rare sections in which a subset of the Nestin-progenitors lining the ventricle expresses PAX3. [file 40478_2014_134_MOESM6_ESM.pdf]

Figure S3

a

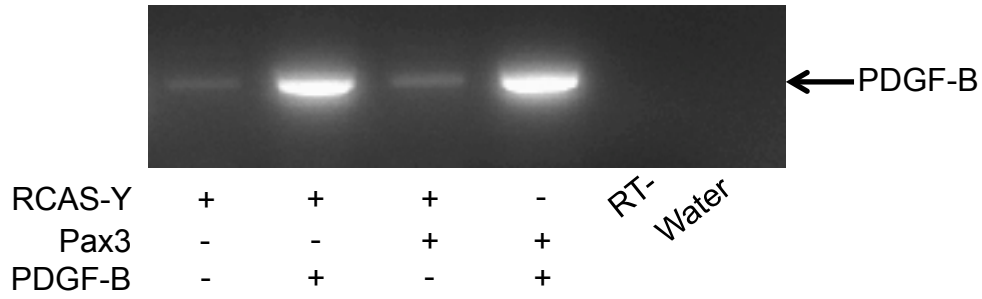

b

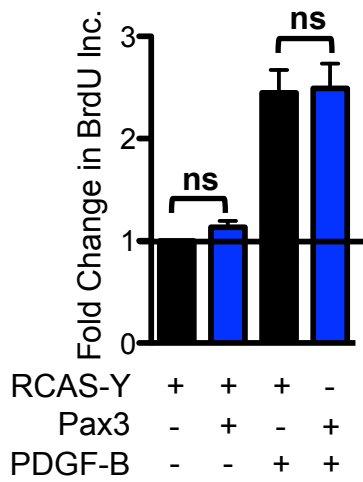

Supplement: Additional file 7: Figure S3. — Normal brainstem progenitors isolated from P3 Ntv-a mice were infected with RCAS-Y, RCAS-Pax3, RCAS-PDGF-B, or RCAS-Pax3 + RCAS-PDGF-B. a mRNA from infected cells was isolated and analyzed for PDGF-B expression by RT-PCR. PCR primers for PDGF-B amplify endogenous mouse PDGF-B as well as the human RCAS-PDGF-B. b BrdU incorporation of normal brainstem progenitors from Ntv-a P3 mice infected with the RCAS viruses as indicated below the graph (RCAS-Y vector was used to control for the total amount of virus). Data is represented as fold-change over RCAS-Y. [file 40478_2014_134_MOESM7_ESM.pdf]

Figure S4

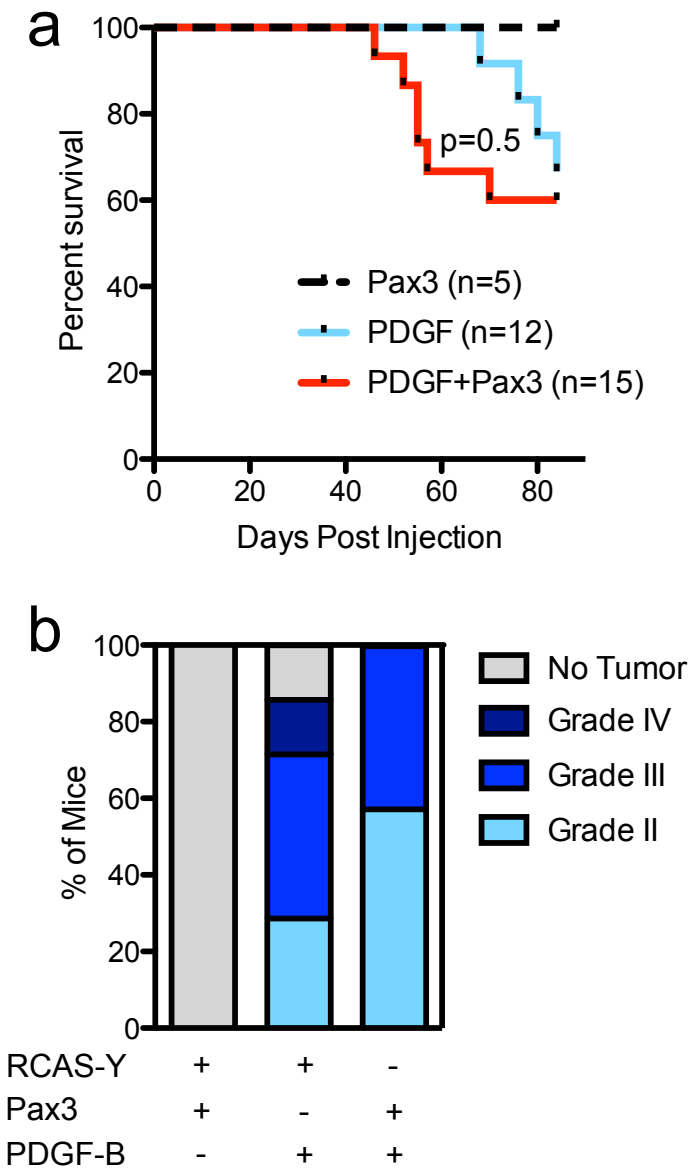

Supplement: Additional file 8: Figure S4. — Ntv-a mice were injected into the cerebral cortex with DF1 cells expressing RCAS-Pax3, RCAS-PDGF-B, or RCAS-PDGF-B + RCAS-Pax3 on P2-4, and monitored for signs and symptoms of brain tumors. a Kaplain-Meier survival curve; p = 0.5, PDGF-B vs. PDGF-B + Pax3. b Mice from the experiment in (a) were sacrificed at the onset of tumor symptoms, or at 12 weeks in the absence of symptoms, and the brains of all mice were analyzed for the presence of glioma using hematoxylin and eosin (H&E) staining, and their tumors graded as described in Materials and Methods. Shown is the percentage of mice in each group with no tumor, grade II, III, and IV glioma. [file 40478_2014_134_MOESM8_ESM.pdf]

Figure S5

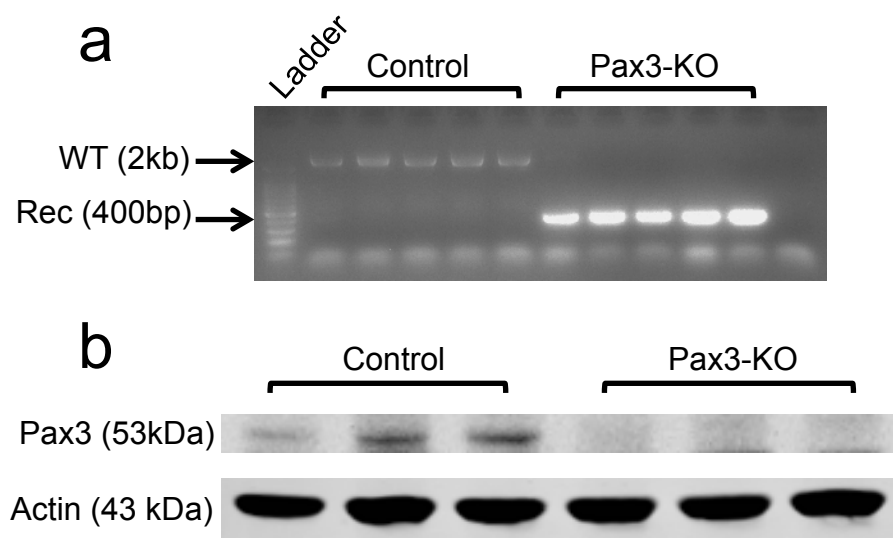

Supplement: Additional file 9: Figure S5. — Deletion of Pax3 in p53-deficient Brainstem Glioma. Ntv-a;p53fl/fl mice (Control) and Ntv-a;p53fl/fl;Pax3fl/fl mice (Pax3-KO) were injected with RCAS-PDGF-B + RCAS-Cre into the brainstem at P2-4. Representative Control and Pax3-KO tumors were analyzed for recombination of the Pax3-floxed allele by PCR of gDNA (a) and for PAX3 protein expression by Western blot (b). [file 40478_2014_134_MOESM9_ESM.pdf]
